# Heart–brain microvascular MRI study: protocol for a multicentre, observational, cohort study in the UK assessing associations between small vessel disease of the heart and brain

**DOI:** 10.1136/bmjopen-2024-088372

**Published:** 2024-12-20

**Authors:** Conor Patrick Bradley, Vanessa Orchard, Robert A Sykes, Gemma McKinley, Alex McConnachie, Paul Donnelly, Jodi Watt, Peter Kellman, Terry Quinn, Natasha Fullerton, Colin Berry

**Affiliations:** 1 British Heart Foundation Glasgow Cardiovascular Research Centre, University of Glasgow, Glasgow, UK; 2 Golden Jubilee National Hospital, West of Scotland Regional Heart and Lung Centre, Glasgow, UK; 3 School of Cardiovascular and Metabolic Health, University of Glasgow, Glasgow, UK; 4 Cardiology, Golden Jubilee National Hospital, Clydebank, UK; 5 Robertson Centre for Biostatistics, University of Glasgow, Glasgow, UK; 6 Queen Elizabeth University Hospital, Glasgow, UK; 7 University of Glasgow, Glasgow, UK; 8 National Heart Lung and Blood Institute, Bethesda, Maryland, USA; 9 Cardiovascular and Medical Sciences, University of Glasgow, Glasgow, UK; 10 University of Glasgow, Golden Jubilee Hospital, Clydebank, West Dunbartonshire, UK

**Keywords:** Ischaemic heart disease, Cognition, Coronary heart disease, Stroke medicine, Dementia

## Abstract

**Introduction:**

Ischaemic heart disease (IHD) and cerebrovascular disease are leading causes of morbidity and mortality worldwide. Cerebral small vessel disease (CSVD) is a leading cause of dementia and stroke. While coronary small vessel disease (coronary microvascular dysfunction) causes microvascular angina and is associated with increased morbidity and mortality. The vascular anatomy of the heart and brain is similar with conduit arteries distributed over the surface of these organs which in turn branch into a network of microscopic penetrating arteries which provide organ perfusion via an end-organ microcirculation. It has also been demonstrated that coronary microvascular dysfunction and CSVD share common vascular risk factors and pathophysiological mechanisms of disease. This has led to a link between the conditions being hypothesised, however, there is an evidence gap clearly demonstrating this relationship. The CorCMR (coronary microvascular angina cardiovascular magnetic resonance imaging) brain imaging study will provide novel insights into the associations between small vessel disease of the heart and brain and related clinical significance.

**Methods and analysis:**

The CorCMR brain imaging study is a prospective, observational, multicentre cohort study including a blinded, central analysis and independent clinical trials unit; a prespecified study nested within the CorCMR trial. We will enrol patients with anginal symptoms who have undergone invasive coronary angiography which has demonstrated no obstructive coronary artery disease. The participants will then undergo brain MRI (to detect CSVD) immediately followed by a quantitative stress perfusion cardiac MRI (to detect coronary microvascular dysfunction). Participants will also undergo neurocognitive testing. The objectives of the study are to assess the prevalence of MRI features of CSVD in patients with angina and no obstructive coronary artery disease; to assess the association between coronary microvascular dysfunction and CSVD and to assess the association between CSVD and cognition.

**Ethics and dissemination:**

The CorCMR study is approved by the UK National Research Ethics Service (Reference 20/WS/0159). Findings will be disseminated through peer-reviewed publications. All patients provided written informed consent.

**Trial registration number:**

ClinicalTrials.gov ID NCT04805814.

**STRENGTHS AND LIMITATIONS OF THIS STUDY**

- Simultaneous imaging of the heart and brain will provide novel insights into the association between heart and brain small vessel disease.
- Prospective multicentre enrolment and large sample size.
- Contemporary quantitative MRI methods for detecting small vessel disease.
- Blinded analysis and an independent statistical analysis undertaken by a clinical trials unit.
- MRI scanning at 1.5 Tesla provides reduced spatial and temporal resolution compared with higher magnetic field strengths.

## Introduction

 Ischaemic heart disease (IHD) and cerebrovascular disease are leading causes of morbidity and mortality worldwide.^1^,^3^ These conditions have a growing clinical and health economic impacts with population ageing.

### Cerebral small vessel disease

Cerebral small vessel disease (CSVD) is a condition which is associated with ageing and has been demonstrated to be universally present in individuals by the age of 90 years.^4^

CSVD has important clinical implications and may present clinically as lacunar stroke or cognitive impairment. CSVD is the cause of a quarter of all acute ischaemic strokes^5^ and almost half of dementia.^6^

The term CSVD describes the vascular abnormalities affecting the perforating cerebral arterioles, capillaries and venules. There are different forms of CSVD, the most common being arteriosclerosis. This condition affects the deep penetrating arterioles (diameter <200 µm) and is characterised by concentric hyaline thickening resulting in fibrosis of the arteriolar wall and subsequent depletion of vascular smooth muscle cells.^7^ Amyloid-related CSVD is caused by mural deposition of amyloid-beta peptide (Aβ) within small leptomeningeal and cortical arteries. Amyloid-beta deposition is an important cause of dementia and is the key pathophysiological driver in 90% of cases of Alzheimer’s disease.^8^

MRI remains the most sensitive imaging modality for detecting CSVD. The MRI features of CSVD are well described and include: (1) lacunes/lacunar infarcts (2) white matter hyperintensities, (3) microbleeds, (4) pathological enlargement of perivascular spaces, (5) microinfarcts, (6) cerebral atrophy. The presence of these features can be used to estimate the burden of CSVD through the calculation of a CSVD score,^9^ which in turn has been demonstrated to correlate with the clinical presentation and outcomes of the patient.^10^ Therefore, brain MRI with CSVD scoring presents an opportunity to identify patients with subclinical CSVD, who may in turn benefit from stratified intensive preventative therapy prior to developing clinical disease.

### Coronary microvascular dysfunction

IHD is one of the leading causes of morbidity and mortality worldwide. The most common clinical presentation of chronic IHD is angina, and the management of angina is largely focused on the detection and treatment of obstructive, flow-limiting atherosclerotic coronary artery disease.^11^ Despite this, more than half of all patients referred for invasive coronary angiography for the investigation of stable angina do not have obstructive coronary artery disease.^12^ Multiple studies have demonstrated that these patients have a condition called microvascular angina.^13^,^15^ Microvascular angina is a condition caused by pathological abnormalities of the coronary small vessels (coronary microvascular dysfunction). It is a common, but under-recognised condition which is associated with an increased risk of major adverse cardiovascular events,^16 17^ persistent anginal symptoms,^18^ impaired quality of life^19^ and considerable health resource utilisation due to recurrent hospitalisations and repeat invasive angiograms.^16^

In the past, coronary microvascular dysfunction often went undiagnosed, however, there have been significant advances in diagnostic methods to assess coronary microvascular function.

The most commonly used methods include invasive functional coronary angiography with acetylcholine testing, stress cardiac MRI, stress Positron Emmision Tomography (PET) and transthoracic Doppler angiography.

Each diagnostic test has advantages and disadvantages, and contemporary international guidelines recommend that the test choice should be guided by local availability and expertise.^20^

While invasive coronary function testing with angiography is still considered to be the gold standard, there have been significant advances in non-invasive imaging techniques in recent years including the development of^20^ automated, pixel-wise quantitative mapping of myocardial perfusion by cardiac MRI for the detection of coronary microvascular dysfunction. This allows for the quantification of myocardial blood flow (measured in mL/min/g tissue) at both stress and resting conditions. This in turn allows for the calculation of myocardial perfusion reserve (a marker of coronary microvascular function), in a similar way to PET.^21^,^23^ Both myocardial blood flow and myocardial perfusion reserve have been demonstrated to be independent predictors of adverse cardiovascular outcomes.^24^

This technique has several advantages over invasive methods including lower cost, better safety profile and lack of ionising radiation. In addition, it has been validated against invasive coronary function testing and PET,^22 25^ and has been shown to have high sensitivity (95%) and specificity (90%).^26^

### Relationship between heart and brain SVD

The vascular anatomy of the heart and brain is similar with conduit arteries distributed over the surface of these organs which in turn branch into a network of microscopic penetrating arteries that provide organ perfusion via an end-organ microcirculation.

It has also been demonstrated that coronary microvascular dysfunction and CSVD share common vascular risk factors (eg, hypertension, ageing and dyslipidaemia) and pathophysiological mechanisms of disease (eg, vasospasm, microemboli, inflammation, neurohormonal dysfunction and atherosclerosis).

This has led to a link between the conditions being hypothesised, however, there is an evidence gap clearly demonstrating this relationship.

## Methods and analysis

### The coronary microvascular angina cardiovascular magnetic resonance imaging (CorCMR) trial

CorCMR is a prospective observational cohort study and a nested double-blind, randomised, controlled clinical trial, which will clarify if routine stress perfusion cardiac MRI reclassifies the final diagnosis in patients with angina with non-obstructive coronary arteries (ANOCA), and whether this diagnostic and management strategy improves symptoms, quality of life and health economic outcomes. A full study design of the nested, randomised control trial has been published previously.^27^ It is summarised below:

Diagnostic study: Stress perfusion cardiac MRI will be used to assess the prevalence of coronary microvascular dysfunction and clinically significant incidental findings (eg, aortic stenosis, cardiomyopathy, lung cancer) in patients with ANOCA. The primary outcome is the between-group difference in the reclassification rate of the initial diagnosis based on invasive angiography versus the final diagnosis after cardiac MRI.

Nested randomised controlled trial: Participants will be randomised to inclusion (intervention group) or exclusion (control group) of myocardial blood flow to inform the final diagnosis. The primary outcome of the clinical trial is the mean within-subject change in the Seattle Angina Questionnaire summary score at 6 months.

### The CorCMR brain imaging study

In the brain imaging arm, all participants will also undergo brain MRI at 1.5 Tesla, and neurocognitive testing, to assess the relationship between coronary microvascular function, CSVD and cognitive function.

The pathophysiology and epidemiology of obstructive atherosclerotic cardiovascular disease, including in the heart and brain, have been intensively investigated. However, much less is known about the associations between small vessel disease in the heart and brain.

### Aim

The aim of the study is to provide novel insights into the associations between small vessel disease in the heart and brain and related clinical significance. The specific aims include:

1. To prospectively assess the prevalence of CSVD in patients with clinically suspected ANOCA following recent (<3 months) diagnostic investigations.
2. To investigate the relationships between CSVD and coronary microvascular dysfunction.
3. To assess the relationships between CSVD and cognitive function, participant characteristics and outcomes in the longer term.

### Hypothesis

We hypothesise that there is a link between coronary microvascular dysfunction, CSVD and cognitive function. Specific hypotheses of the trial include:

1. In patients with ANOCA confirmed by invasive coronary angiography assessments, CSVD is prevalent.
2. Coronary microvascular dysfunction quantified by stress cardiac MRI (as measured by myocardial blood flow (mL/min/g)) is independently associated with CSVD.
3. The presence of CSVD on MRI is associated with impaired cognitive function in patients with ANOCA.

### Study design

A prospective, observational, multicentre cohort study including a blinded, central analysis and independent clinical trials unit; a prespecified study nested within the CorCMR trial.

### Objectives

The objectives include:

1. Assess the prevalence of MRI features of CSVD in patients with ANOCA.
2. Assess the association between coronary microvascular dysfunction and MRI features of CSVD.
3. Assess the association between myocardial blood flow (mL/min/g) and MRI features of CSVD.
4. Assess the association between MRI features of small vessel disease and cognition as measured by the Montreal Cognitive Assessment (MOCA).
5. Assess the association between MRI features of small vessel disease and patient characteristics.
6. Assess the association between MRI features of small vessel disease and long-term health outcomes.

### Implementation

**Figure 1:**
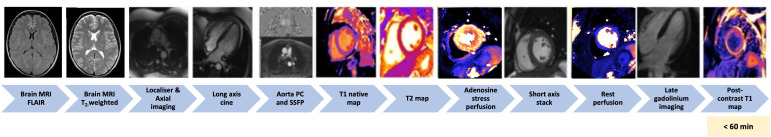
MRI brain–heart protocol.

The study will enrol patients with ANOCA who have undergone invasive coronary angiography at three different hospitals, within the 3 months preceding enrolment period. Following informed consent, participants will be invited to return for MRI scans at a single reference imaging centre (NHS Golden Jubilee Hospital). All participants will undergo a brain MRI scan at 1.5 Tesla immediately followed by a quantitative stress perfusion cardiac MRI (figures1  2).

**Figure 2:**
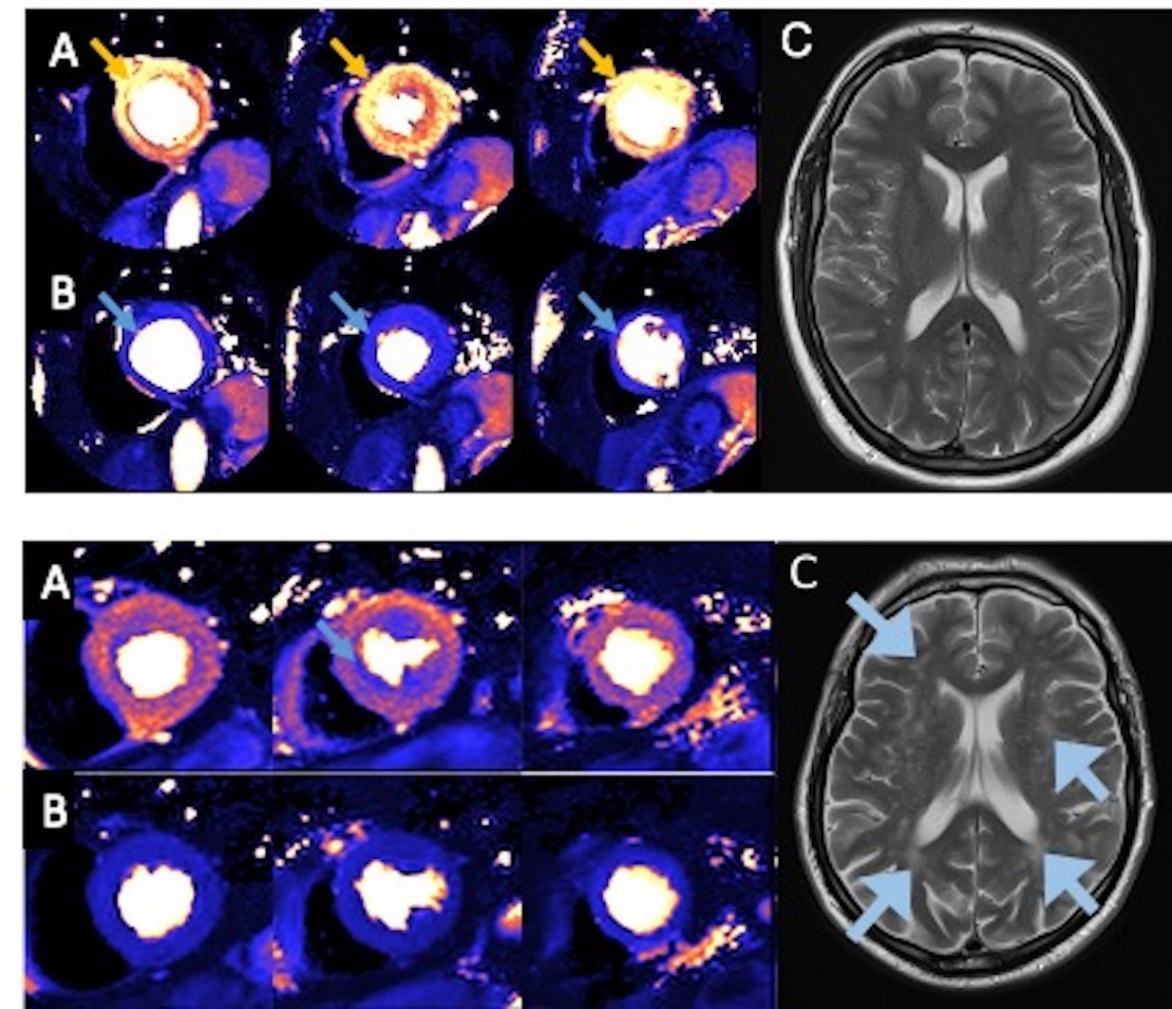
Two clinical cases from coronary microvascular angina cardiovascular magnetic resonance imaging (CorCMR) Trial. Top: normal cardiac and brain MRI scans. A 42-year-old man with anginal symptoms and no obstructive coronary artery disease on invasive coronary angiography. He was enrolled into the CorCMR clinical trial. (**A**) Normal stress MRI (bright orange, arrow) with no perfusion defects (ie, no residual blue colour). (**B**) Cardiac MRI shows normal myocardial blood flow at rest (uniform dark blue, arrow). (**C**) Normal MRI brain scan with no evidence of cerebral small vessel disease (CSVD). Bottom: small vessel disease in the heart and brain. A 59-year-old woman with anginal symptoms and no obstructive coronary artery disease on invasive coronary angiography. She was enrolled into the CorCMR clinical trial. (**A**) Stress MRI showing circumferential subendocardial perfusion defect (dark blue, arrow) that contrasts with the normal hyperaemic blood flow (bright orange) elsewhere in the heart. Diagnostic of coronary microvascular dysfunction/microvascular angina. (**B**) Cardiac MRI shows normal blood flow at rest (upper panel – dark blue, arrow). (**C**) Brain MRI showing extensive periventricular and subcortical white matter hyperintensities (blue arrows) in keeping with CSVD. In summary, this participant has microvascular angina and brain imaging features of CSVD.

### Setting

The study is based in Central and West Scotland, including Glasgow and other urban and rural areas accommodating 2.5 million people. The participating sites include three hospitals in West and Central Scotland (NHS Golden Jubilee Hospital, University Hospital Hairmyres and University Hospital Ayr). The sites include a regional cardiothoracic centre, a large urban hospital and a district general hospital.

### Study population

Outpatients referred for invasive coronary angiography for investigation of angina will be eligible to participate. A Participant Information Sheet for the study will be provided to patients before and/or after coronary angiography.

### Eligibility

Participants can be enrolled in the study after coronary angiography, when obstructive atherosclerosis in the main epicardial coronary arteries (≥2.5 mm lumen diameter) has been excluded. Enrolment may occur up to 3 months after the coronary angiogram.

#### Inclusion criteria

- Age ≥18 years.
- Symptoms of angina informed by the Rose Angina questionnaire.
- Coronary angiography ≤3 months with a plan for medical management.

#### Exclusion criteria

- Obstructive coronary artery disease, that is, a stenosis >70% in a single segment or 50–70% in two adjacent segments in an artery >2.5 mm, or fractional flow reserve (FFR) ≤0.80.
- A diagnosis that would explain the angina, for example, anaemia, aortic stenosis and hypertrophic cardiomyopathy.
- Contraindication to contrast-enhanced cardiac MRI, for example, estimated Glomerular Filtrate Rate (eGFR) <30 mL/min/1.73m^2^.
- Contraindication to intravenous adenosine, that is, severe asthma; long QT syndrome; second- or third-degree AV block and sick sinus syndrome; lack of informed consent.

### MRI protocol and analysis

The Study Information Sheet and Consent form will be provided to patients before or after the standard care coronary angiogram. After the angiogram, eligibility will be reconfirmed. Following written informed consent, study participants will be invited to return for an MRI within 3 months.

#### MRI brain

MRI will be performed at 1.5 Tesla (Siemens MAGNETOM Avanto, Erlangen, Germany), using a standardised MRI protocol. Brain MRI will be performed prior to stress cardiac MRI. Brain MRI will include 3D fluid-attenuated inversion recovery (FLAIR) (1 mm thick, whole head wherever feasible) acquisition, axial T_2_-weighted TSE sequences, susceptibility-weighted imaging (SWI) and diffusion-weighted (DWI), as per standard local protocols.

#### MRI brain analysis

The MRI brain scan will be reported by a Consultant Neuroradiologist, who will be blind to the cardiac MRI findings. The report will document the presence or absence of incidental findings (eg, cerebral aneurysms, brain tumours). Each MRI brain scan will be assessed by the Staff Neuroradiologist for the presence of small vessel disease (periventricular white matter hyperintensities (WMH), subcortical/deep WMH, lacunes/lacunar infarcts, deep grey nuclei and lobar microhaemorrhages). WMH will be graded based on modifications of standardised scales accepted for clinical trial purposes (Fazekas scale for white matter lesions).^28^ The total MRI brain small vessel disease burden will be scored using validated scoring systems (table 1) and on a nominal scale, adapted from peer-reviewed studies.^9 29^ In addition, each scan will also be scored for trial purposes as to the presence of vascular disease in the form of established territorial, cortical and cerebellar infarcts, and as to the presence of acute/subacute ischaemia (table 2). Brain volume in relation to a patient’s age will also be assessed (table 3).

**Table 1:** Total small vessel disease score and categories

MRI feature	Visual assessment	Definition	Score
Lacunes	International consensus definition^34^	>1 Lacune	1 point
Microbleeds	International consensus definition^34^	>1 Microbleed	1 point
Perivascular spaces	Semiquantitative scale^35^	Moderate to severe perivascular spaces in basal ganglia	1 point
White matter hyperintensities (WMH)	Fazekas scale^28^	Periventricular WMH Fazekas 3 (extending into deep white matter) and/or deep WMH Fazekas 2–3 (confluent or early confluent)	1 point
Total score (0–4)			

**Table 2:** Standardised score for the presence of cerebral small vessel disease (CSVD) burden

MRI feature	Assessment	Score
Periventricular white matter hyperintensities (WMH)	Absent/minor	0
	Mild to moderate	1
	Marked	2
Subcortical/deep WMH	Absent/minor	0
	Mild to moderate	1
	Marked	2
Lacunes/lacunar infarcts	Absent	0
	Present	1
Deep grey nuclei and lobar microhaemorrhages	Absent	0
	Present	1
Total score (0–6)		

**Table 3:** Brain atrophy assessment

Brain atrophy assessment	Score
Maintained brain volume	0
Age-appropriate involutional change	1
Brain volume loss in excess of what would be expected for age	2

#### MRI heart

The cardiovascular 1.5 Tesla MRI protocol has been described in detail.^27^ The imaging protocol will include localisers, cine imaging for cardiovascular dimensions and function, aortic cine and flow sequences, T1-mapping and T2 mapping precontrast, adenosine stress- and rest-perfusion imaging, late gadolinium enhancement imaging and postcontrast myocardial T1 mapping.

### Questionnaires and follow-up

The MOCA will be administered by research staff to assess participants’ cognition.^30^ The questionnaire will be administered at baseline and 12 months. Follow-up assessments for adverse events will be performed by the clinical research staff by telephone or in person (eg, outpatient clinic review), as appropriate. Follow-up contact will occur at 6 monthly intervals until the last patient has achieved a minimum of 12 months of follow-up. Follow-up in the longer term (ie, ≥3 years) will be supported by electronic record linkage with central government health records.

### Statistical analyses

The CorCMR study has a comprehensive statistical analysis plan that governs all statistical aspects of the study authored by the trial statistician and has been published previously.^27^ The statistical analysis plan includes the prespecified brain arm that is designed to assess associations between MRI features of small vessel disease and myocardial blood flow measured by stress cardiac MRI.

#### Sample size calculation

To assess an association between myocardial blood flow (mL/min/g tissue) and features of CSVD, for 90% power, 5% significance and a minimal clinically significant correlation of 0.3, 110 subjects will be needed. Recruitment is expected to significantly exceed this, as a sample size of 250 is required for the main CorCMR study.

### Trial management and governance

#### Trial management

The study will be conducted according to observational (Strengthening the Reporting of Observational Studies in Epidemiology),^31^ Good Clinical Practice^32^ and Consolidated Standards of Reporting Trials guidelines.^33^

#### Ethics and dissemination

The CorCMR study is approved by the UK National Research Ethics Service (Reference 20/WS/0159). Findings will be disseminated through peer-reviewed publications. All patients provided written informed consent.

#### Sources of funding

The study is funded by the British Heart Foundation (PG/19/28/34310; RE/18/6134217). The Chief Scientist Office funds the cardiac MRI scans. The funders have no other involvement in the study.

#### Registration

The ClincalTrials.gov registration is NCT04805814.

#### Patient and public involvement

The design of this study has been informed by discussions with patients and their families about improving what is known about angina in patients who do not have obstructive coronary disease.

#### Study progress

Participant recruitment and follow-up are currently ongoing.

## Discussion

Our study will provide novel insights into the associations between small vessel disease in the heart and brain and related clinical significance.

### Strengths and limitations

Design attributes include an unselected population of patients with angina with non-obstructive coronary arteries (ANOCA, prospective multicentre enrolment, a relatively large sample size, use of validated patient-reported outcome measures, simultaneous imaging of the heart and brain, contemporary quantitative MRI methods for small vessel disease in these organs, blinded analysis and an independent statistical analysis undertaken by a clinical trials unit. Few studies have investigated vascular disease in the heart and brain, and prior studies have been limited by design, for example, retrospective case selection with a small sample size.

Limitations of the study include MRI scanning at 1.5 Tesla provides reduced spatial and temporal resolution compared with higher magnetic field strengths. Furthermore, CMR has limitations including contraindication in certain patient groups, for example, severe renal disease, claustrophobia and implantable devices.

### Clinical implications

The advances in cardiac MRI technology described will be used in combination with brain MRI in the coronary microvascular angina cardiovascular magnetic resonance imaging (CorCMR) trial. The CorCMR brain study is designed and powered to assess the association between MRI features of cerebral small vessel disease (CSVD) and coronary microvascular dysfunction, as measured by quantitative stress perfusion cardiac MRI. It will also assess if there is an association between MRI features of CSVD and cognitive function. This will be one of the largest trials assessing the relationship between heart and brain small vessel disease.

This has important clinical implications as the demonstration of a link between heart and brain small vessel disease presents the opportunity for future research into the development of preventative and therapeutic treatments to target these important conditions.
