# Supplementary material for: Heart–brain microvascular MRI study: protocol for a multicentre, observational, cohort study in the UK assessing associations between small vessel disease of the heart and brain
Source: BMJ Open. 2024 Dec 20;14(12):e088372. doi: 10.1136/bmjopen-2024-088372 (PMC11667430; doi:10.1136/bmjopen-2024-088372)
Supplement: Supplementary file 1 [file bmjopen-2024-088372.reviewer_comments.pdf]

PEER REVIEW HISTORY

BMJ Open publishes all reviews undertaken for accepted manuscripts. Reviewers are asked to complete a checklist review form and are provided with free text boxes to elaborate on their assessment. These free text comments are reproduced below.

ARTICLE DETAILS

Title (Provisional)

The Heart - Brain Microvascular Magnetic Resonance Imaging Study: Protocol for a multicentre, observational, cohort study in the UK assessing associations between small vessel disease of the heart and brain

Authors

Bradley, Conor Patrick; Orchard, Vanessa; Sykes, Robert A; McKinley, Gemma; McConnachie, Alex; Donnelly, Paul; Watt, Jodi; Kellman, Peter; Quinn, Terry; Fullerton, Natasha; Berry, Colin

VERSION 1 - REVIEW

**Reviewer**	**1**
**Name**	**Song, Yi**
**Affiliation**	**The First Affiliated Hospital of Zhengzhou University**
**Date**	**18-Jun-2024**
**COI**	**No potential conflict of interest was reported.**

This protocol mainly describes the CorCMR brain imaging study, which aims to explore the association between heart and brain vascular disease. The study design includes multicenter prospective recruitment of patients with non-obstructive coronary artery disease, the use of advanced quantitative MRI methods to detect vessel disease in these organs, simultaneous imaging of the heart and brain, and blinded and independent statistical analysis performed by the clinical trial unit. The strengths of the study include providing new insights into the association between heart and brain vessel disease, multicenter recruitment and large sample size, and the use of advanced MRI methods.

1. This paragraph lacks content on the pathology, symptoms, and significance of CMD on P8 (Line 97). I recommend the authors to provide a more detailed description.

2. This paragraph is too simple, the logic and purpose are unclear, and the two should be correlated on P8 (Line 109). I recommend the authors to provide a more detailed description.

3. What are clinically significant incidental findings on P9 (Line 125)? I recommend the authors should list them in detail.

4. What does “inclusion (intervention group) or exclusion (control group) of myocardial blood flow” mean on P9(Line 130)?

5. A large number of full spellings without abbreviations. For example: Cerebral small vessel disease (CSVD).

6. The abbreviated expression of an MRI imaging is repetitive. For example: MRI Imaging should be MRI.

7. It is not stated what the relationship is in Aim 2) on P10. The relationship between CSVD and cognition has been widely studied, how long is the long-term result, and what are the characteristics of the participants in Aim 3) on P10?

8. What does “± physiology” mean on P10 (Line 149), Does this mean any physiological examination? I recommend the authors should list them in detail. ？

9. What does “related” mean in Hypothesis 2 on P10, what is the relationship, and any certain indicators of cardiac MRI?

10. The conclusion of Hypothesis 3 is too general and has been widely studied on P10. What is the innovation in the conclusion of Hypothesis 3?

11. Is “blinded, central analysis and independent clinical trials unit” consistent with "blinded independent central reviews" on P10 (Line 156)?

12. Should the objective be clearer, and more specific? I recommend revising the objective.

13. There are no exclusion criteria in the brain disease history. I recommend exclusion criteria should be added to.

14. The authors perform a Brain MRI using a cardiac protocol on P13(Line 219). I recommend revising it.

15. What are the incidental findings in the brain examination on P13(Line 226)? Please list them in detail.

16. I checked the two criteria for evaluating CSVD are inconsistent with the STRIVE scale mentioned in the secondary outcome item 22 which is published as follows

(https://classic.clinicaltrials.gov/ct2/show/NCT04805814) in the clinical trial registration on P13(Line 232). STRIVE has been updated to STRIVE-2, and the evaluation of cerebral small vessel disease on Brain MR images has also been modified. Please verify them. If the old standard is used, please fully explain the reason.

17. Please explain the calculation process of Brain volume in detail on P14(Line 237).

18. The questionnaires are conducted at baseline and 12 months in manuscript. During the follow-up process, should an MRI be performed again on P14(Line 251)? I recommend at

least performing a Brain MRI to clarify the progression of small vessel disease and the questionnaire should be conducted at baseline, 6 months, and 12 months.

19. I recommend taking into account the sample size and the number of lost follow-ups on P15(Line 276).

20. There seems to be no content related to Progress to date (one of the requirements of the journal protocol).

21. T2w of the Brain MRI is shown, but all the sequences of the cardiac MRI are shown in Figure 1. If the authors want to show the small vessel ischaemic damage in T2w, why not add the FLAIR MRI? Please list the reasons.

22. I recommend the description of adenosine in the figure 2 legend should be placed in the method section. The last sentence is inappropriate, this study is still in progress, and there is no result to support this statement in Figure 2.

23. The Cerebral-Coronary Connection (C3) study is described in much detail on P17(Line 309), but there is no corresponding reference (Maybe: PMCID: PMC9825810). At the same time, it is necessary to explain the difference between this study protocol and the reference above.

24. Do the microvessels of the coronary arteries of the heart be called small vessels on P18(Lines 325, 338)? What is the definition of the microvessels of the coronary arteries of the heart and the small vessels in the brain?

25. The overall text of the discussion needs to be improved, the content of the relevant research should be simplified and the innovation of this study should be highlighted in comparison with previous studies.

26. Please place the references in the corresponding content, not in the subheading on P8 (line 109).

27. The title of some references is inconsistent in capitalization: the first letter of the sentence is capitalized/the first letter of each word is capitalized.

28. Punctuation issues. For example, P7 Line71 (1-3) should be revised to [1-3], and P9 Line The 1),2),3) in Aim should be corrected to 1.2.3. I recommend the authors revise them.

**Reviewer**	**2**
**Name**	**Beijnink, Casper**
**Affiliation**	**Radboudumc, Cardiology**
**Date**	**01-Aug-2024**
**COI**	**none**

In this manuscript, Bradley et al. describe the rationale and design of the Heart-Brain microvascular resonance imaging study. Their aim is to study the shared characteristics and overlap between coronary and cerebral microvascular disease and to study associations between findings on CMR and brain MRI. It seems plausible that microvascular cerebral and coronary disease would co-exist but evidence pointing in this direction is currently lacking.

Strengths of the study will be its prospective nature and thorough imaging protocols.

I believe that the manuscript could be improved, by addressing the following issues:

Major

1. Please elaborate on quantitative stress perfusion CMR for the detection of microvascular CAD. What is it expected yield in patients without epicardial stenoses? More importantly, what are the exact sensitivity and specificity of quantitative stress perfusion CMR for microvascular disease as compared to the gold standard (invasive measurements?) Please further elaborate in the paper as it may be a limitation of the study design.

2. Do the authors expect that their findings may change clinical practice? If not, are their findings expected to stimulate further research into the association between microvascular cerebral and coronary artery disease? The paper would benefit from a (potential) clinical implications section.

Minor

1. Page 4 line 52; Participants with also undergo neurocognitive testing. Please change 'with' to will

2. It seems obvious that the addition of a complete CMR study will significantly improve the diagnostic work-up. Therefore, why did the authors choose the nested randomized controlled trial design? Would it not be better to just show in how many cases information from CMR changes the diagnosis?

3. Line 117; From here the authors begin to describe the study methods. Please move this section from introduction to methods. Please also describe first what a nested randomized controlled trial is, to those readers unfamiliar with it. As it stands, it appears that not all patients underwent brain MRI and CMR (which is untrue as I understand from the methods section).

4. Methods and analysis, line 141: This way of describing the study aims and hypothesis is unconventional and seems more suitable for a study protocol. For readability, the manuscript may benefit from a conventional descriptive style of writing.

5. Methods, line 262; please specify which endpoints were adjudicated as significant, if relevant.

6. Methods, line 276, sample size calculation. Did the authors choose 80% power and aim to include 49 subjects? Please specify

7. Manuscript title (line 1); It would be better to write rationale with an e at the end.

VERSION 1 - AUTHOR RESPONSE

Reviewer 1:

1. This paragraph lacks content on the pathology, symptoms, and significance of CMD on P8 (Line 97). I recommend the authors to provide a more detailed description.

RESPONSE: Thank you for this comment. We have now provided a more detailed description. We hope the following statement clarifies this:

Page 7, Lines 111-121

"Angina is the most common symptom of chronic ischaemic heart disease (IHD), and the management of angina is often focused on the detection and treatment of flow-limiting atherosclerotic coronary artery disease[1]. However, most patients referred for an invasive coronary angiogram for the investigation of stable anginal symptoms do not have obstructive coronary artery disease[2]. Multiple studies have shown that the majority of these patients have coronary small vessel disease[3–5]. This is a condition referred to as microvascular angina caused by coronary microvascular dysfunction. Coronary microvascular dysfunction is not benign and is associated with increased risk of major adverse cardiovascular events[6,7], persistent anginal symptoms[8], impaired quality of life[9], and considerable health resource utilization due to recurrent hospitalizations and repeat invasive angiograms[6]."

2. This paragraph is too simple, the logic and purpose are unclear, and the two should be correlated on P8 (Line 109). I recommend the authors to provide a more detailed description.

RESPONSE: Thank you for this comment. The purpose of this paragraph is to highlight the anatomical similarities and shared pathophysiological mechanisms of disease in both the heart and brain microcirculation. We have updated this to provide a more detailed description, however, the word limit means we cannot fully explore this in any more depth. We have updated the text as follows:

Page 8, Lines 148-156

“The vascular anatomy of the heart and brain is similar with conduit arteries distributed over the surface of these organs. These arteries then branch into a microscopic network of penetrating arteries which provide organ perfusion via an end-organ microcirculation. In addition, common pathogenic risk factors have been identified for the development of both coronary microvascular dysfunction and CSVD, e.g., diabetes, hypertension, and increasing age. Furthermore, shared pathophysiological mechanisms such as atherosclerosis, inflammation, neurohormonal dysfunction, vasospasm, and microemboli, contribute to small vessel disease in both organs. This has led to a link between the conditions being hypothesized, however, there is an evidence gap clearly demonstrating this relationship.”

3. What are clinically significant incidental findings on P9 (Line 125)? I recommend the authors should list them in detail.

RESPONSE: Thank you for this comment. We have now updated the text to include the most common clinically significant incidental findings found on cardiac MRI. The text now reads:

Page 9, Lines 202-204

“In the diagnostic study, stress perfusion cardiac MRI will be used to assess the prevalence of coronary microvascular dysfunction and clinically significant incidental findings (e.g. aortic stensosis, cardiomyopathy, lung cancer) in patients with ANOCA.”

Unfortunately, the word limit prevents us from providing a detailed list of incidental findings, however, we have attached the study protocol which has an explanation regarding incidental findings. It states:

“Incidental findings

An incidental finding in a patient undergoing an imaging examination may be defined as “a finding that has potential health or reproductive importance which is discovered in the course of conducting research, but is beyond the aims of the study” [10]. The overall approach to incidental findings has been reviewed[11,12], and guidelines have been developed [13] and evaluated [14]. In CorCMR, the primary aim for undertaking the MRI scan is to assess myocardial perfusion. Contemporary CMR is a multi-parametric examination that provides information on heart structure, function, blood flow, tissue characterization and extra-cardiac features. During the standard care reporting process, abnormalities that are disclosed beyond

the blood flow component will be considered incidental and their clinical significance will be classified and actioned by the reporting clinicians, as appropriate.

An actionable incidental finding is defined by the Actionable Reporting Work Group (ARWG) of the American College of Radiologists (ACR) [12]. Actionable findings are considered clinically significant. A list of incidental findings of potential pathological significance has been pre-determined in line with contemporary standards of care . They are findings that require communication within minutes (category 1), within hours (category 2), or within days (category 3). A stable finding that was previously known and appropriately communicated may not require additional nonroutine communication despite the severity of the disease process (category 4). The ACR group notes the list of actionable findings is not definitive.

Wolf and colleagues categorised the clinical significance of incidental findings, and related implications for disclosure/actioning, into three categories: strong net benefit, possible net benefit, and unlikely net benefit [10].

- **Strong net benefit** - a condition likely to be life threatening, or a condition likely to be grave that can be avoided or ameliorated. This includes information about a condition likely to be life threatening, even if it cannot be treated. These are incidental findings whose disclosure is likely to offer markedly more benefit than burden to the research participant. This is an actionable finding.
- **Possible net benefit** - may offer more benefit than burden to the research participant. An incidental finding in this category reveals a health condition that cannot be avoided or ameliorated. Researchers may reveal this type of incidental finding to show respect for the informational needs and preferences of the research participant, even though the information is not likely to change the participant's own clinical course. In the view of Wolf, researchers are not obligated to offer this information.
- **Unlikely net benefit** – disclosures offers more burden than benefit and therefore are not relevant to disclose. This would be a non-actionable incidental finding.

Table. Classification of incidental finding for the primary outcome (actionable, yes/no)

ACR Criteria	Wolff classification	Actionable (Primary outcome)
1 – 3	Strong net benefit	Yes
2, 3	Possible net benefit	Yes
4	Unlikely net benefit	No

4. What does "inclusion (intervention group) or exclusion (control group) of myocardial blood flow" mean on P9(Line 130)?

RESPONSE: This refers to the nested randomised controlled trial where patients are randomised to the intervention arm (in which myocardial blood flow results are used to inform the diagnosis) or control arm (in which myocardial blood flow results are not used, and diagnosis is based on coronary angiography). We have clarified this by including a reference to the full study design paper for the main study:

Page 9, Lines 207-211

"In the nested randomized controlled trial participants will be randomized to inclusion (intervention group) or exclusion (control group) of myocardial blood flow to inform the final diagnosis. The primary outcome of the clinical trial is the mean within-subject change in the Seattle Angina Questionnaire summary score at 6 months. A full study design of the nested, randomized control trial has been published previously[15]."

5. A large number of full spellings without abbreviations. For example: Cerebral small vessel disease (CSVD).

RESPONSE: The manuscript has been reviewed to reduce the number of full spellings and replace these with the appropriate abbreviations throughout.

6. The abbreviated expression of an MRI imaging is repetitive. For example: MRI Imaging should be MRI.

RESPONSE: The term MRI imaging has been removed and replaced by MRI throughout the manuscript.

7. It is not stated what the relationship is in Aim 2) on P10. The relationship between CSVD and cognition has been widely studied, how long is the long-term result, and what are the characteristics of the participants in Aim 3) on P10?

RESPONSE: Thank you for this comment. At present it is not understood what the relationship is between CSVD and coronary microvascular dysfunction. Thus, the purpose of this study is to investigate this further.

As detailed in the follow-up section of the manuscript, follow-up will be for a minimum of 12 months but is expected to be >3 years via electronic record linkage.
Participant characteristics refers to patient baseline characteristics collected as part of the trial (e.g. age, sex, co-morbidities, medications etc).

8. What does "± physiology" mean on P10 (Line 149), Does this mean any physiological examination? I recommend the authors should list them in detail.？
RESPONSE: This referred to coronary physiology testing (i.e. coronary function testing). We have now removed this as we appreciate it is not relevant to the brain imaging study and may be confusing to a non- cardiology audience.

9. What does "related" mean in Hypothesis 2 on P10, what is the relationship, and any certain indicators of cardiac MRI?
RESPONSE: Thank you for this comment. We have updated the text to clarify this:

Page 10, Lines 235-236
"Coronary microvascular dysfunction quantified by stress cardiac MRI (as measured by myocardial blood flow (ml/min/g)) is independently associated with CSVD."

10. The conclusion of Hypothesis 3 is too general and has been widely studied on P10. What is the innovation in the conclusion of Hypothesis 3?
RESPONSE: Thank you for this comment. This has not been widely studied in an NAOCA population. We have therefore updated the text to clarify this point:

Page 10, Lines 237-238
"The presence of CSVD on MRI is associated with impaired cognitive function in patients with ANOCA."

11. Is "blinded, central analysis and independent clinical trials unit" consistent with "blinded independent central reviews" on P10 (Line 156)?
RESPONSE: Thank you for this comment. Yes, these two statements are consistent. All scans are analysed centrally by a blinded, independent neuroradiologist, and statistical analysis is undertaken by an independent clinical trials unit.

12. Should the objective be clearer, and more specific? I recommend revising the objective.

RESPONSE: Thank you for this comment. As this is a largely unstudied area of medicine, the purpose of the study is largely hypothesis generating and to inform and stimulate future research. Therefore, following discussion with key stakeholders, particularly patient advocate groups for patients with coronary microvascular dysfunction, it was felt that broad objectives were important in order to investigate this potential link with CSVD.

13. There are no exclusion criteria in the brain disease history. I recommend exclusion criteria should be added to.

RESPONSE: Thank you for this comment. There are no specific exclusion criteria for patients with a brain disease history. The only exclusion criteria specific to this would be a contraindication to MRI, which is already included in the manuscript.

14. The authors perform a Brain MRI using a cardiac protocol on P13(Line 219). I recommend revising it.

RESPONSE: Thank you for this comment. This error has now been corrected.

15. What are the incidental findings in the brain examination on P13(Line 226)? Please list them in detail.

RESPONSE: Thank you for this comment. We have now updated the text to include the most common incidental findings. We hope the following text clarifies this:

Page 14, Lines 320-321

"The report will document the presence or absence of incidental findings (e.g. cerebral aneurysms, brain tumours)."

As with the response to point 3, Unfortunately, the word limit prevents us from providing a detailed list of incidental findings, however, we have attached the study protocol which has an explanation regarding incidental findings

16. I checked the two criteria for evaluating CSVD are inconsistent with the STRIVE scale mentioned in the secondary outcome item 22 which is published as follows

(https://classic.clinicaltrials.gov/ct2/show/NCT04805814) in the clinical trial registration on P13(Line 232). STRIVE has been updated to STRIVE-2, and the evaluation of cerebral small vessel disease on Brain MR images has also been modified. Please verify them. If the old standard is used, please fully explain the reason.

RESPONSE: Thank you for this comment. Unfortunately, STRIVE-2 was only published in July 2023, meaning recruitment, scanning of patients, and reporting of brain MRI scans had already started for our study prior to the updated standard being released. Therefore, to ensure consistent scanning and reporting for all patients, we have adhered to the older STRIVE-1 guidance for all patients in the study.

17. Please explain the calculation process of Brain volume in detail on P14(Line 237).

RESPONSE: Thank you for this comment. Brain volume is not quantitatively measured, but is visually assessed by an expert consultant neuroradiologist as detailed in table 3 as either (1) Maintained brain volume (2) Age-appropriate involutional change, or (3) Brain volume loss in excess of what would be expected for age.

18. The questionnaires are conducted at baseline and 12 months in manuscript. During the follow-up process, should an MRI be performed again on P14(Line 251)? I recommend at least performing a Brain MRI to clarify the progression of small vessel disease and the questionnaire should be conducted at baseline, 6 months, and 12 months.

RESPONSE: Thank you for this comment. We agree that in an ideal study there would be serial brain MRI to assess progression of CSVD. However, our study is charity funded by the British Heart Foundation, and financial restraints mean that there is not the budget to pay for serial imaging for patients. We accept that this is a limitation of the study.

19. I recommend taking into account the sample size and the number of lost follow-ups on P15(Line 276).

RESPONSE: Thank you for this comment. We have now updated this section:

Page16, Lines 378-382

"*Sample size calculation*

To assess for an association with myocardial blood flow (ml/min/g tissue) and features of CSVD, for 90% power, 5% significance and a minimal clinically significant correlation of 0.3 then 110 subjects will be needed. Recruitment is expected to significantly exceed this as a sample size of 250 is required for the main CorCMR study."

20. There seems to be no content related to Progress to date (one of the requirements of the journal protocol).

RESPONSE: Thank you for this comment. We have now added the following section:

Page 17, Lines 408-409

"*Study Progress*

Participant recruitment and follow-up is currently ongoing."

21. T2w of the Brain MRI is shown, but all the sequences of the cardiac MRI are shown in Figure 1. If the authors want to show the small vessel ischaemic damage in T2w, why not add the FLAIR MRI? Please list the reasons.

RESPONSE: Thank you for this comment. Figure 1 has now been updated to include a FLAIR MRI image:

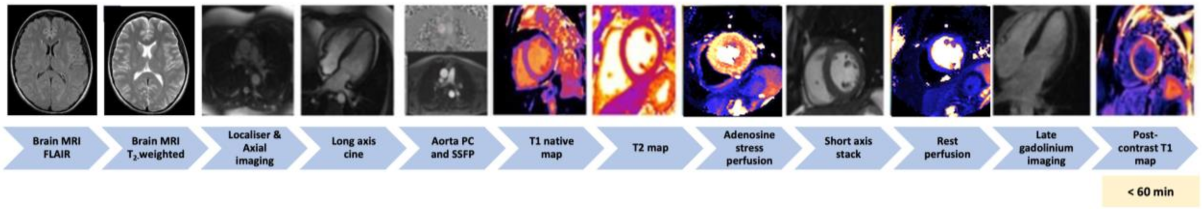

22. I recommend the description of adenosine in the figure 2 legend should be placed in the method section. The last sentence is inappropriate, this study is still in progress, and there is no result to support this statement in Figure 2.

RESPONSE: Thank you for this comment. The description of the action of adenosine has been removed. This is instead referenced in the methods section, where the main study design protocol paper is referenced. The last sentence has also been removed.

23. The Cerebral-Coronary Connection (C3) study is described in much detail on P17(Line 309), but there is no corresponding reference (Maybe: PMCID: PMC9825810). At the same time, it is necessary to explain the difference between this study protocol and the reference above.

RESPONSE: The discussion has been shortened on the advice of the editors, and reference to the Cerebral-Coronary Connection (C3) study has now been removed.

24. Do the microvessels of the coronary arteries of the heart be called small vessels on P18(Lines 325, 338)? What is the definition of the microvessels of the coronary arteries of the heart and the small vessels in the brain?

RESPONSE: Thank you for this comment. Small vessel is a descriptive term that is commonly used in the literature. We believe it is helpful to used shared terminology for both heart and brain small vessel disease. This is the nomenclature used in other trials and review articles[16].

The coronary microvasculature, namely the pre-arterioles and arterioles, is comprised of a complex system of micro-vessels <500 μm in diameter. Cerebral small vessels have been histologically defined by a size of <1 mm in vessel diameter, most often between 50 and 400μm.

25. The overall text of the discussion needs to be improved, the content of the relevant research should be simplified and the innovation of this study should be highlighted in comparison with previous studies.

RESPONSE: Thank you for this comment. We have now revised the discussion to predominantly focus on the strengths and limitations of the study, as well as the innovation of the study as suggested.

26. Please place the references in the corresponding content, not in the subheading on P8 (line 109).

RESPONSE: Thank you for this comment. This has now been corrected.

27. The title of some references is inconsistent in capitalization: the first letter of the sentence is capitalized/the first letter of each word is capitalized.

RESPONSE: The capitalisation used in the titles of all references has been taken directly from the individual journals in which they have been published, and as displayed on PubMed.

Unfortunately, whilst this does result in inconsistencies, it is the most true to the original publications.

28. Punctuation issues. For example, P7 Line71 (1-3) should be revised to [1-3], and P9 Line The 1),2),3) in Aim should be corrected to 1.2.3. I recommend the authors revise them.

RESPONSE: These punctuation issues have now been corrected.

Reviewer 2:

Major

1. Please elaborate on quantitative stress perfusion CMR for the detection of microvascular CAD. What is it expected yield in patients without epicardial stenoses? More importantly, what are the exact sensitivity and specificity of quantitative stress perfusion CMR for microvascular disease as compared to the gold standard (invasive measurements?) Please further elaborate in the paper as it may be a limitation of the study design.

RESPONSE: Thank you for this comment. We have added additional information to the manuscript to answer the questions raised. We have elaborated more on quantitative stress perfusion CMR for the detection of coronary microvascular dysfunction. In particular, we have highlighted the high sensitivity and specificity compared with invasive measurement of coronary function. We hope the following statement clarifies this:

Page 7-8, Lines 125-145

"Each technique has strengths and limitations, and contemporary guidelines recommend that the test choice should be guided by local availability and expertise[17].

Contemporary advances in non-invasive imaging include automated, pixel-wise quantitative mapping of myocardial perfusion by cardiac MRI for the detection of coronary microvascular dysfunction. This method generates pixel-encoded maps of myocardial blood flow (ml/min/g tissue), which are acquired during vasodilator (hyperemic) stress and resting conditions. Myocardial perfusion reserve is calculated in a similar way to PET[18–20]. The novel cardiac MRI method has been validated against invasive measures of coronary function and PET[19,21], and has been shown to have high sensitivity (95%) and specificity (90%)[22].

Furthermore, myocardial blood flow and myocardial perfusion reserve measured using this method are independent predictors of adverse cardiovascular outcomes[23].”

Furthermore, in patients without epicardial stenoses, the expected yield of detecting coronary microvascular dysfunction has been shown to vary amongst trials but is consistently >50% of all patients. We have therefore added the following statement, with supporting references, to clarify this:

Page 7, Lines 113-116
“However, most patients referred for an invasive coronary angiogram for the investigation of stable anginal symptoms do not have obstructive coronary artery disease[2]. Multiple studies have shown that the majority of these patients have coronary small vessel disease[3–5].”

2. Do the authors expect that their findings may change clinical practice? If not, are their findings expected to stimulate further research into the association between microvascular cerebral and coronary artery disease? The paper would benefit from a (potential) clinical implications section.

RESPONSE: Thank you for this comment. Whilst we do not expect this study to change current clinical practice, we do expect it to inform and stimulate future research. We agree that the paper would benefit from a clinical implications sections, and have therefore added this to the discussion:

Page 18, Lines 425-434
“*Clinical Implications*
The advances in cardiac MRI technology described, will be used in combination with brain MRI in the CorCMR trial. The CorCMR brain study is designed and powered to assess the association between MRI features of CSVD and coronary microvascular dysfunction, as measured by quantitative stress perfusion cardiac MRI. It will also assess if there is an association between MRI features of CSVD and cognitive function. This will be one of the largest trials assessing the relationship between heart and brain small vessel disease.
This has important clinical implications as demonstration of a link between heart and brain small vessel disease presents the opportunity for future research into the development of preventative and therapeutic treatments to target these important conditions.”

Minor

1. Page 4 line 52; Participants with also undergo neurocognitive testing. Please change 'with' to will

RESPONSE: This error has now been corrected.

2. It seems obvious that the addition of a complete CMR study will significantly improve the diagnostic work-up. Therefore, why did the authors choose the nested randomized controlled trial design? Would it not be better to just show in how many cases information from CMR changes the diagnosis?

RESPONSE: Thank you for your comment. Whilst we agree that the addition of a complete CMR study will improve the diagnostic work-up, it has not been shown that this in turn improves clinical outcomes. The purpose of the nested randomised trial is to investigate if accurately diagnosing coronary microvascular dysfunction actually improves outcomes for patients and reduces symptom burden (as measured by Seattle Angina Questionnaire). In order for quantitative stress perfusion CMR to be adopted into routine clinical practice there needs to be evidence of this improvement in outcomes. This has never been investigated before, but our nested randomised trial design allows for this.

3. Line 117; From here the authors begin to describe the study methods. Please move this section from introduction to methods. Please also describe first what a nested randomized controlled trial is, to those readers unfamiliar with it. As it stands, it appears that not all patients underwent brain MRI and CMR (which is untrue as I understand from the methods section).

RESPONSE: Thank you for this comment. We have now moved this section from the introduction to methods.

We have also provided a fuller explanation of the nested randomised controlled trial design, with a reference to the dedicated trial design paper:

Page 9, Lines 207-211

"In the nested randomized controlled trial participants will be randomized to inclusion (intervention group) or exclusion (control group) of myocardial blood flow to inform the final diagnosis. The primary outcome of the clinical trial is the mean within-subject change in the

Seattle Angina Questionnaire summary score at 6 months. A full study design of the nested, randomized control trial has been published previously[15].”

All patients in the study will undergo both a cardiac and brain MRI. We hope the following statement clarifies this:

Page 9, Line 214
“pants will undergo a brain MRI scan at 1.5 Tesla immediately followed by a quantitative stress perfusion cardiac MRI.”

4. Methods and analysis, line 141: This way of describing the study aims and hypothesis is unconventional and seems more suitable for a study protocol. For readability, the manuscript may benefit from a conventional descriptive style of writing.
RESPONSE: Thank you for this comment. We have added additional text to hopefully make the writing style more descriptive. However, the specific aims and hypotheses have also been retained, in keeping with the style of protocol articles previously published in this journal.

Page 10, Lines 221-238
“Aim
The aim of the study is to provide novel insights into the associations between small vessel disease in the heart and brain and related clinical significance. The specific aims include:

1) To prospectively assess the prevalence of CSVD in patients with clinically suspected ANOCA following recent (<3 months) diagnostic investigations.
2) To investigate the relationships between CSVD and coronary microvascular dysfunction.
3) To assess the relationships between CSVD and cognitive function, participant characteristics, and outcomes in the longer term.

Hypothesis
We hypothesise that there is a link between coronary microvascular dysfunction, CSVD, and cognitive function. Specific hypotheses of the trial include:

1. In patients with ANOCA confirmed by invasive coronary angiography assessments, CSVD is prevalent.
2. Coronary microvascular dysfunction quantified by stress cardiac MRI (as measured by myocardial blood flow (ml/min/g)) is independently associated with CSVD.

3. The presence of CSVD on MRI is associated with impaired cognitive function in patients with ANOCA."

5. Methods, line 262; please specify which endpoints were adjudicated as significant, if relevant.

<u>RESPONSE:</u> Thank you for this comment. We agree that this section is not clear. The clinical events committee will assess major adverse cardiovascular events. This text has now been amended to clarify this:

Page 15-16, Lines 360-367

"Major adverse cardiovascular events were assessed by a Clinical Event Committee according to a pre-specified charter. This committee will be blind to the baseline data and randomized groups. The committee will be independent of the investigators, funder, and sponsor."

6. Methods, line 276, sample size calculation. Did the authors choose 80% power and aim to include 49 subjects? Please specify

<u>RESPONSE:</u> Thank you for this comment. Our original text was not clear, and has now been amended to clarify. It reads as follow:

Page 16, Lines 379-382

"To assess for an association with myocardial blood flow (ml/min/g tissue) and features of CSVD, for 90% power, 5% significance and a minimal clinically significant correlation of 0.3 then 110 subjects will be needed. Recruitment is expected to significantly exceed this as a sample size of 250 is required for the main CorCMR study."

7. Manuscript title (line 1); It would be better to write rationale with an e at the end.

<u>RESPONSE:</u> Thank you for this comment. The manuscript title has now been updated at the request of the editors. The manuscript title is now:

"The Heart - Brain Microvascular Magnetic Resonance Imaging Study: Protocol for a multicentre, observational, cohort study in the UK assessing associations between small vessel disease of the heart and brain"

<u>References</u>

1 Knuuti J, Wijns W, Saraste A, *et al.* 2019 ESC Guidelines for the diagnosis and management of chronic coronary syndromes. *Eur Heart J*. 2020;41:407–77. doi: 10.1093/eurheartj/ehz425

2 Patel MR, Peterson ED, Dai D, *et al.* Low Diagnostic Yield of Elective Coronary Angiography. *New England Journal of Medicine*. 2010;362:886–95. doi: 10.1056/NEJMoa0907272

3 Ford TJ, Stanley B, Good R, *et al.* Stratified Medical Therapy Using Invasive Coronary Function Testing in Angina. *J Am Coll Cardiol*. 2018;72:2841–55. doi: 10.1016/j.jacc.2018.09.006

4 Camici PG, Crea F. Coronary Microvascular Dysfunction. *New England Journal of Medicine*. 2007;356:830–40. doi: 10.1056/NEJMra061889

5 Sara JD, Widmer RJ, Matsuzawa Y, *et al.* Prevalence of Coronary Microvascular Dysfunction Among Patients With Chest Pain and Nonobstructive Coronary Artery Disease. *JACC Cardiovasc Interv*. 2015;8:1445–53. doi: 10.1016/j.jcin.2015.06.017

6 Jespersen L, Hvelplund A, Abildstrom SZ, *et al.* Stable angina pectoris with no obstructive coronary artery disease is associated with increased risks of major adverse cardiovascular events. *Eur Heart J*. 2012;33:734–44. doi: 10.1093/eurheartj/ehr331

7 Taqueti VR, Solomon SD, Shah AM, *et al.* Coronary microvascular dysfunction and future risk of heart failure with preserved ejection fraction. *Eur Heart J*. 2018;39:840–9. doi: 10.1093/eurheartj/ehx721

8 Jespersen L, Abildstrøm SZ, Hvelplund A, *et al.* Persistent angina: highly prevalent and associated with long-term anxiety, depression, low physical functioning, and quality of life in stable angina pectoris. *Clin Res Cardiol*. 2013;102:571–81. doi: 10.1007/s00392-013-0568-z

9 Tavella R, Cutri N, Tucker G, *et al.* Natural history of patients with insignificant coronary artery disease. *Eur Heart J Qual Care Clin Outcomes*. 2016;2:117–24. doi: 10.1093/ehjqcco/qcv034

10 Wolf SM, Lawrenz FP, Nelson CA, *et al.* Managing incidental findings in human subjects research: analysis and recommendations. *J Law Med Ethics*. 2008;36:219–48, 211. doi: 10.1111/j.1748-720X.2008.00266.x

11 Bluemke DA, Liu S. Imaging in Clinical Trials. *Principles and Practice of Clinical Research*. Elsevier 2012:597–617.

12 Rodrigues JCL, Lyen SM, Loughborough W, *et al.* Extra-cardiac findings in cardiovascular magnetic resonance: what the imaging cardiologist needs to know. *J Cardiovasc Magn Reson*. 2016;18:26. doi: 10.1186/s12968-016-0246-1

13 Larson PA, Berland LL, Griffith B, *et al.* Actionable findings and the role of IT support: report of the ACR Actionable Reporting Work Group. *J Am Coll Radiol*. 2014;11:552–8. doi: 10.1016/j.jacr.2013.12.016

14 Farrah TE, Webb DJ, Dhaun N. Retinal fingerprints for precision profiling of cardiovascular risk. *Nat Rev Cardiol*. 2019;16:379–81. doi: 10.1038/s41569-019-0205-2

15 Bradley CP, Orchard V, McKinley G, *et al.* The coronary microvascular angina cardiovascular magnetic resonance imaging trial: Rationale and design. *Am Heart J*. 2023;265:213–24. doi: 10.1016/J.AHJ.2023.08.067

16 Berry C, Sidik N, Pereira AC, *et al.* Small-vessel disease in the heart and brain: Current knowledge, unmet therapeutic need, and future directions. *J Am Heart Assoc*. 2019;8:11104. doi: 10.1161/JAHA.118.011104

17 Gulati M, Levy PD, Mukherjee D, *et al.* 2021 AHA/ACC/ASE/CHEST/SAEM/SCCT/SCMR Guideline for the Evaluation and Diagnosis of Chest Pain: A Report of the American College of Cardiology/American Heart Association Joint Committee on Clinical Practice Guidelines. *Circulation*. 2021;144:e368–454. doi: 10.1161/CIR.0000000000001029

18 Kellman P, Hansen MS, Nielles-Vallespin S, *et al.* Myocardial perfusion cardiovascular magnetic resonance: optimized dual sequence and reconstruction for quantification. *Journal of Cardiovascular Magnetic Resonance*. 2017;19:43. doi: 10.1186/s12968-017-0355-5
19 Engblom H, Xue H, Akil S, *et al.* Fully quantitative cardiovascular magnetic resonance myocardial perfusion ready for clinical use: a comparison between cardiovascular magnetic resonance imaging and positron emission tomography. *J Cardiovasc Magn Reson*. 2017;19:78. doi: 10.1186/s12968-017-0388-9
20 Hsu L-Y, Jacobs M, Benovoy M, *et al.* Diagnostic Performance of Fully Automated Pixel-Wise Quantitative Myocardial Perfusion Imaging by Cardiovascular Magnetic Resonance. *JACC Cardiovasc Imaging*. 2018;11:697–707. doi: 10.1016/j.jcmg.2018.01.005
21 Knott KD, Camaioni C, Ramasamy A, *et al.* Quantitative myocardial perfusion in coronary artery disease: A perfusion mapping study. *Journal of Magnetic Resonance Imaging*. 2019;50:756–62. doi: 10.1002/jmri.26668
22 Rahman H, Scannell CM, Demir OM, *et al.* High-Resolution Cardiac Magnetic Resonance Imaging Techniques for the Identification of Coronary Microvascular Dysfunction. *JACC Cardiovasc Imaging*. 2021;14:978–86. doi: 10.1016/j.jcmg.2020.10.015
23 Knott KD, Seraphim A, Augusto JB, *et al.* The Prognostic Significance of Quantitative Myocardial Perfusion: An Artificial Intelligence Based Approach Using Perfusion Mapping. *Circulation*. 2020;CIRCULATIONAHA.119.044666. doi: 10.1161/CIRCULATIONAHA.119.044666

VERSION 2 - REVIEW

**Reviewer**	**2**
**Name**	**Beijnink, Casper**
**Affiliation**	**Radboudumc, Cardiology**
**Date**	**21-Oct-2024**
**COI**	

The authors are commended with their revised manuscript. All my questions and remarks have been answered to satisfaction.
